# The Regional Burden of Parkinson's Disease in Kazakhstan 2014–2021: Insights From National Health Data

**DOI:** 10.1155/padi/4317554

**Published:** 2025-05-01

**Authors:** Ruslan Akhmedullin, Arnur Gusmanov, Gulnur Zhakhina, Byron Crape, Temirgali Aimyshev, Yuliya Semenova, Gaziz Kyrgyzbay, Abduzhappar Gaipov

**Affiliations:** ^1^Department of Medicine, Nazarbayev University School of Medicine, Astana, Kazakhstan; ^2^Department of Functional Diagnostics, RSE Medical Centre Hospital of the President's Affairs Administration of the Republic of Kazakhstan, Astana, Kazakhstan

**Keywords:** comorbidity, DALY, epidemiology, mortality, Parkinson's disease

## Abstract

**Background:** This study explores the burden of Parkinson's disease (PD) in Kazakhstan, the largest country in Central Asia, a region where data on neurological disorders are notably sparse.

**Methods:** Utilizing data from Kazakhstan's Unified National Electronic Health System during 2014–2021, the study investigates the epidemiology, disability-adjusted life years (DALYs), and survival outcomes in a cohort of PD patients. The authors employed Cox proportional hazards regression models and Kaplan–Meier analysis, alongside sensitivity analyses, to assess the impact of demographic factors, hypertension, and the Charlson Comorbidity Index (CCI) on survival.

**Results:** The study cohort included 10,125 patients, revealing a tenfold increase in PD prevalence during the study period. Mortality rates varied significantly, with the highest rates observed in the eldest age group (137.05 per 1000 person-years). PD contributed to a loss of 156.12 DALYs per 100,000 population, primarily driven by years of life lost. The analysis identified an increased risk of all-cause mortality among males (adjusted hazard ratio (aHR) 1.6; 1.5–1.8), older individuals (aHR 1.05; 1.04–1.06), those with higher CCIs, and individuals of Kazakh ethnicity. Interestingly, patients with comorbid hypertension had a higher probability of survival (aHR 0.67; 0.60–0.73).

**Conclusion:** This study is the first of its kind in Central Asia to examine the burden of PD using a large-scale outpatient registry. The findings underscore the need for targeted interventions to address the growing burden of PD, particularly among males and ethnic Kazakhs. Additionally, further research is needed to explore the inverse association between hypertension and survival in the PD cohort.

## 1. Introduction

Parkinson's disease (PD) is the second most prevalent progressive neurodegenerative disorder, affecting 2%–3% of individuals aged ≥ 65 years. It is one of the most rapidly growing neurodegenerative conditions worldwide and contributes significantly to disability [1, 2]. PD is characterized by the degeneration of dopaminergic neurons in the substantia nigra of the midbrain and the development of Lewy bodies, which are distinctive pathological features of PD [2, 3]. The International Parkinson and Movement Disorder Society (MDS) defines the core feature of PD as motor Parkinsonism, which is characterized by bradykinesia along with rest tremor or rigidity [4]. At present, age is the most influential risk factor for PD with potential associations with various air pollutants [5]. Although clinical PD diagnosis is characterized by bradykinesia and key motor features, molecular pathogenesis involves diverse pathways, suggesting that multiple triggering mechanisms contribute to PD development [1, 6].

PD is characterized as chronic and nonfatal, and its rising prevalence is associated with an aging population. Global disability and mortality indices for PD have rapidly increased, doubling in prevalence over the past 25 years [7]. Today, neurological disorders are the leading cause of disability worldwide, and PD is one of the fastest-growing neurodegenerative conditions among them. Relying on estimates from the Global Burden of Disease (GBD) study [8], in 2017, the prevalence of PD was 8.5 million (95% CI 7.0–10.1). The most recent GBD study [9] evaluated 5.5 million disability-adjusted life years (DALYs) and 1.2 million years lived with disability (YLDs) for PD. The number of patients with PD is expected to rise as the population life expectancy grows, resulting in an increase in the number of patients with advanced PD. To address this burden, there is a need for better preventive strategies and effective treatment for PD.

Currently, there is a need for more epidemiological studies, especially in areas where data are limited, to analyze temporal trends and their driving factors [5, 10]. Little is known about the burden of PD in Central Asia. Although there is an abundance of data available from high-income countries, there is a lack of comprehensive data from developing countries, especially Central Asia. This study aimed to evaluate the burden of PD in Kazakhstan, the largest country in Central Asia, a region where data on neurological disorders are notably sparse.

## 2. Materials and Methods

### 2.1. Study Design and Population

This study utilized large-scale administrative healthcare data from the Unified National Electronic Health System (UNEHS) spanning over a period of 2014–2021. UNEHS started in 2003, was established in 2014, and made available medical claims from various data sources, which were implemented throughout the country. Further clarification of the database and methodology can be found in previous studies [11–13]. PD events were identified on the basis of the International Classification of Diseases (ICD). Raw records were extracted from the database of the “G20” ICD codes from the outpatient dispensary registry. The cases were confirmed using standard procedures based on the local and MDS guidelines, including physical examination, magnetic resonance imaging, transcranial ultrasound dopplerography, and the UK Parkinson's Disease Society Brain Bank criteria [4, 14]. The initial data comprised 15,898 patient records. Thorough data cleaning resulted in 10,125 records (Figure S1), each with a unique population registry number (RPN ID). The population size of Kazakhstan and its growth rate were obtained from the local Bureau of National Statistics [15].

### 2.2. Exposure and Covariates

Individual patient data encompassed essential demographic information, such as date of birth, sex, ethnicity, residence, ICD-10 diagnosis, PD diagnosis dates, and date of death with the corresponding cause, if applicable. The study entry date was January 1, 2014, and the endpoint of the follow-up period was June 30, 2021. Using the dispensary registry, we determined the date of each RPN ID diagnosis. Age at disease diagnosis was determined as the difference between the diagnosis date and date of birth and was stratified into five categories: < 50 years, 50–59 years, 60–69 years, 70–79 years, and individuals aged 80 years and above. As more than 50 ethnicities were present, we grouped them into three ethnicities: Kazakhs, Russians, and others. Data for ethnicity covariates were missing from 91 observations (0.90%). Considering their low prevalence, they were recoded into the “other” group.

### 2.3. Outcome Assessment

The crude incidence and prevalence were calculated by dividing the new and living cases by the total population at the end of each consecutive year. For age-specific incidence and prevalence, these numbers were divided by the cohort size in the corresponding age groups. The prevalence analysis was calculated cumulatively, and participants were excluded from the analysis only in case of death. Time at risk was determined as the difference between the study entry date and the date of death or the end of follow-up, whichever came first. Charlson Comorbidity Index (CCI) was a 4-level scale without age adjustment [16]. Years of life lost (YLLs) were calculated by multiplying the death counts in an age group by the remaining life expectancy, taken from the GBD standard life table [17]. Regarding the YLDs, a prevalence-based approach was used, where the index was multiplied by the disability weight (DW). DW varied according to the severity levels of PD (e.g., mild, moderate, and severe). As the severity was not available in the UNEHS, the average of the three DWs was taken [18]. DALYs were then calculated as the sum of YLLs and YLDs.

### 2.4. Statistical Analysis and Methods

The incidence and prevalence rates were expressed per 100,000 people in each age category. Mortality rate was expressed as per 1000 person-years. Data management, cleaning, and overall analyses were performed using STATA (Version 18). A map of PD prevalence was constructed using the QGIS 3.32.1. Data were summarized as percentages for categorical variables, as mean and standard deviation (SD) for values following normal distribution, and as median and interquartile range (IQR) for nonsymmetric distribution. Exploratory and predictive data analysis and Cox proportional hazards regression analyses were employed to obtain crude and adjusted hazard ratios (aHRs) on incident cases. Kaplan–Meier survival analysis was performed using log-rank and Wald's tests to calculate the failure function and statistically significant differences in survival by age, sex, comorbidities, ethnicity, and CCI with all-cause mortality. Coefficient estimates and variance inflation factor (VIF) were used to detect multicollinearity, and the model fit was tested using the Schoenfeld test. A series of sensitivity analyses was performed to test the credibility of the findings. *p* values were two-sided and reported as statistically significant at *p* < 0.05. Given the retrospective nature of the study, the Institutional Review Ethics Committee of Nazarbayev University waived the need for informed consent and approved the study (NU-IREC 490/18112021). This study was performed in accordance with the “STROBE” guidelines [19]. The data that support the findings of this study are available on request from the corresponding author. The data are not publicly available due to privacy and ethical restrictions.

## 3. Results

### 3.1. Sociodemographic Characteristics

Patient characteristics are summarized in Table 1. During the study period, PD diagnosis was observed more in females (60.7%) than males (39.3%). The majority of patients were aged between 60 and 69 years (35.5%), with the percentage being the lowest among those aged 50 years and younger (6.2%). The mean age at the time of PD diagnosis was 66.9 years (±10.7), while the median duration of the disease was 2.5 years (IQR 1.3–4.2). Among the study cohort, Kazakhs made up more than half of the population with PD (50.9%), followed by Russians (30.8%) and “other” ethnicities (18.3%). Coexisting hypertension was present in 57.9% of patients with PD.

### 3.2. Epidemiological and Mortality Estimates

The incidence rate increased slightly within the observation period, especially for those aged ≥ 80 years, increasing to 56% by 2021 (Figure S2). However, prevalence estimates have grown substantially, in particular, from 4.2 in 2014 to 44.1 in 2021 per 100,000 (Figure S2). In terms of age-specific prevalence, the lowest and highest rates were observed in the youngest and the eldest groups, respectively. The mortality rate in the entire PD cohort was 60.4 (95% CI 57.6–63.3) per 1000 person-years, with the highest rate for those aged 80 years, and gradually decreasing for each consecutive age group.

**Figure 1:**
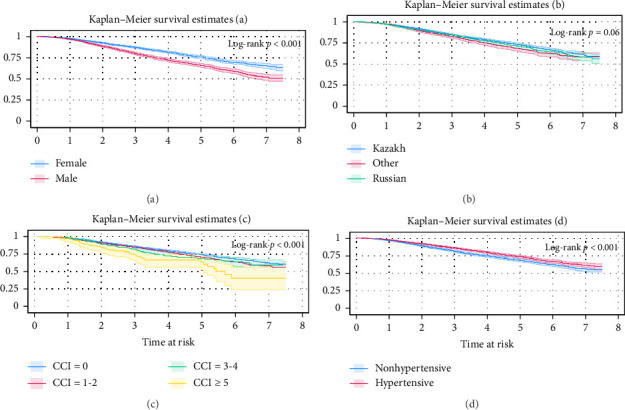
Survival probability by sex, ethnicity, CCI, and hypertension.

In Kaplan–Meier survival analysis, the probability of survival was in male patients than in female patients, and in those of Kazakh ethnicity compared to “other” ethnicities, including Russian (Figure 1 and Table 2). Additionally, survival decreased with higher CCI scores. Patients with comorbid hypertension have a higher chance of survival. Finally, the results indicated an increased risk of all-cause mortality among the male sex (aHR = 1.62 (95% CI 1.48–1.79)), older age (aHR = 1.05; 95% CI 1.04–1.06), and elevated CCI levels (CCI = 1-2 (aHR = 1.26; 1.14–1.40), CCI = 3-4 (aHR = 1.30; 1.06–1.56), CCI ≥ 5 (aHR = 2.10; 1.56–2.80); however, hypertensive patients (aHR = 0.66; 0.60–0.73), Russian (aHR = 0.84; 95% CI 0.75–0.94), and others (aHR = 0.97; 95% CI 0.87–1.11) showed a lower risk when compared to female sex, younger age, CCI = 0, Kazakh ethnicity, and normotensive patients (Table 2).

### 3.3. DALY

**Figure 2:**
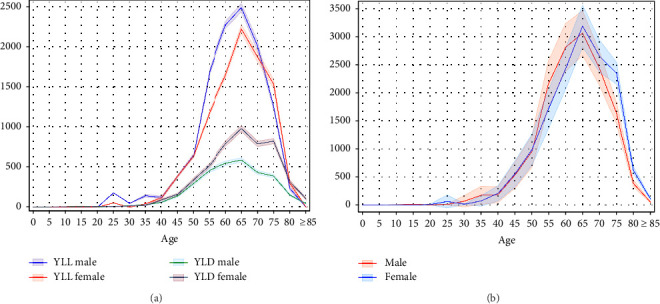
Sex-adjusted years of life lost and years lived with disability (a) and disability-adjusted life years (b).

The age- and sex-adjusted YLLs, YLDs, and DALYs in the PD cohort are presented in Figure 2 (Table S1). Throughout the study period, 29,474 DALYs were lost due to PD (156.12 per 100,000). The YLL and YLD values were 21,287 and 8,187, respectively. The highest contribution of both premature deaths and years lost due to disability to DALY is observed at the ages of 65–75 years when YLLs and YLDs are the highest. The total DALYs for men and women were similar (14, 491 and 14, 982, respectively).

### 3.4. Sensitivity Analysis

PD was uncommon in patients aged less than 50 years. Consequently, in the sensitivity analysis for survival, we excluded participants in this age group (Figure S3(A)), and the estimates (aHR) remained unchanged. Similarly, further exclusion of individuals aged over 80 years at the time of study entry did not alter the estimates (Figure S3(A)). The exclusion of age adjustment from the CCI scores showed an increase in estimates solely for CCIs; however, the VIF value suspected substantial multicollinearity (10.92), and the goodness-of-fit test was poor (0.06). Subsequent age-adjustment removal from the CCIs solved this issue (VIF = 2.01; Schoenfeld test = 0.79). Propensity score matching was performed using age, sex, CCIs, and ethnicity as covariates to test the effect of hypertension on survival in 3690 hypertensive patients versus 3690 nonhypertensive patients (Figure S4). The estimates remained unchanged (log-rank test *p* < 0.001). Finally, the study findings suggest that COVID-19 did not have a significant impact on survival within the study cohort. The confidence intervals for the mortality rates from 2014 to 2021 were substantially overlapping, indicating that there were no distinct changes in mortality rates during 2020–2021 compared to previous years. This suggests that the effect of COVID-19 on overall mortality in this cohort was minimal (Figure S3(B)).

## 4. Discussion

Neurological disorders are currently the primary cause of disability worldwide, and PD is one of the fastest-growing neurodegenerative conditions. To our knowledge, it is the first of its kind in Kazakhstan to evaluate the burden of PD using a large-scale outpatient registry. In the study cohort, PD was more frequent in females, the majority were Kazakhs, and most of the cases were diagnosed in individuals aged 60–69. More than half of the patients had comorbid hypertension, which is inversely associated with survival. Although the incidence rate increased during the observation period, there was a slight decline in 2020. Despite the female predominance in prevalent PD, the contribution of males to DALYs was substantially greater.

### 4.1. Epidemiological and Mortality Estimates

Although the incidence rate increased during the observation period, there was a slight decline in 2020. These estimates appeared to be affected by the COVID-19 pandemic. Although the lowest index across the study period was in April 2020, a month after the declaration of the stringent lockdown [20], it began to rise, and by November 2020, it reached the highest mark. Overall, the results showed an increase in PD incidence, prevalence, and mortality over 8 years, which is consistent with worldwide trends [5, 9, 21]. Our study showed that PD prevalence was substantially lower than the global estimates [5], but similar to the regional estimates [22]. However, unlike our approach, this study used ICD-10 codes G21 (secondary Parkinsonism) and G22 (Parkinsonism in diseases classified elsewhere) in addition to G20 (PD). Nevertheless, our estimates are significantly lower than those for neighboring Russia [23] and former Soviet Union members Estonia [24] and Azerbaijan [25]. Notably, these studies did not provide details regarding the employed ICD-10th codes; for this reason, discrepancies might be explained by case identification. On the other hand, an epidemiological study from another Soviet Union country from (Ukraine) [26] which employed only “G20” reported estimates that were aligned with our indices. A previous review [27] that utilized data from a doctoral thesis showed that the prevalence of PD in Kazakhstan is 65 per 100,000 individuals. However, the reference was not available, and we failed to reach its methodological approaches. Furthermore, the validity of PD diagnosis remains unsatisfactory [28]. Accuracy has not significantly improved in recent decades, particularly during the early stages of the disease. These findings suggest that a large proportion of patients may have been misdiagnosed and/or unregistered. However, the increasing trends across the period are consistent with world estimates [9, 21]. The burden of PD is less pronounced in individuals younger than 65 years. Therefore, the observed growth can be partly attributed to the increasing share of this age group in Kazakhstan's population [10, 29]. This shift in demographic structure may potentially contribute to a rise in incident cases of PD. Interestingly, both the incidence and prevalence of PD are less common in males than in females, while global estimates have reported inverse predominance [5, 30]. The reasons for these are likely multifactorial, which can be explained by variations in access to healthcare or differences in healthcare-seeking behavior, underdiagnosis/misdiagnosis due to differences in symptom presentation, or healthcare utilization. To further understand these associations, it is crucial that future studies concentrate on this topic.

The northern, eastern, and central regions had the greatest prevalence of PD; however, the nearby administrative units showed lower indices (Figure S5). It is worth noting that it is expressed as per 100,000, and owing to the absence of data on age distributions, the geographical prevalence estimates may not accurately reflect the variations between regions. Further studies should explore age-specific prevalence estimates.

According to the findings of this study, more than half of the participants were diagnosed with hypertension. Furthermore, its role in survival was “protective,” which raises concerns regarding the credibility of estimates. Thus, hypertension was associated with a lower risk of death, although its prevalence in the study was higher than that in the general population of Kazakhstan [31], but similar to those reported in the PD cohort [32, 33]. The natural history of hypertension in patients with autonomic failure remains unknown. End-organ damage is linked to plasma renin activity, which only manifests after chronic, untreated essential hypertension. Consequently, low renin levels in autonomic failure have been reported to have a protective effect against hypertension complications [34]. In addition, the use of calcium channel blockers in patients with PD was associated with a reduced risk of death [35]. Nevertheless, the reduction in our study may be explained by confounding factors that were not taken into account or by the presence of complex syndromes that cause this reverse association, which need to be thoroughly explored. These findings might substantially affect the reliability of the data, which is inconsistent with the literature [36], and require caution when interpreting the observed association. However, our mortality rates are within the range reported in the previous studies [37, 38].

### 4.2. DALY

The 2016 GBD study reported the level of standardized YLL rates for each cause and trend since 2006, represented as the annualized rate of change in the age-standardized YLL rate. PD has been one of the two causes of significantly positive rates of change since 2006 [17]. The results of our study are concordant with the literature, and the contributions of YLLs to DALY and DALYs for females were higher [5]. However, unlike the global trends [22], our YLD estimates were conversely oriented, with female patients having a higher burden. This might be related to the higher prevalence in females in our study; however, YLLs were less due to increased mortality in males. The distribution of DALYs among the regions showed a specific pattern that developed concurrently with prevalence rates.

### 4.3. Sensitivity Analysis

Based on sensitivity analyses, our results demonstrated the reliability of the survival estimates. Its consistency remained unchanged even after excluding specific age groups and testing the age-adjustment effects of covariates. However, collinearity tests yielded the best model for the survival estimates. These results confirm the resilience of our conclusions regarding the impact of COVID-19 on survival in the study cohort. Nevertheless, our study lacked information on individual COVID-19 infections. As a result, our estimates are descriptive and should be further explored in future studies with more complete data. Taken together, these suggest the robustness of our findings, as the estimates largely remained unchanged. Nevertheless, the consistency of the reverse estimates for hypertension highlights caution when interpreting its effect on the survival in the PD cohort.

### 4.4. Strengths and Limitations

Our findings are important for several reasons, as follows. First, this is the first study in Central Asia to demonstrate the burden of PD based on nationwide healthcare data. No large studies have evaluated the loss of DALYs in this region. The findings of our study may potentially be extrapolated to other Central Asian countries, considering the common culture, lifestyle, and healthcare model. Moreover, this study demonstrated geographical disparities in the prevalence of PD. This knowledge can lead to changes in health policies and provide direction for policymakers. However, our study had a few limitations. First, the evaluation of disease epidemiology is solely based on secondary data, which does not represent all PD cases in the country. This database lacks information on important risk factors, such as heredity, environmental causes, head trauma, and the presence of Lewy bodies, which are distinct features of PD. Moreover, the DW used for the estimation of the DALY was approximated using severity levels. There was no information on pain intensity, which would allow us to perform a more precise calculation. Ethnicity data were missing from 91 observations. We assumed that missingness did not depend on observed or missing values. Further recoding them into “other” group allowed us to keep the entire cohort for analyses. In addition, considering its low rate (< 1%), we believe that its impact on the survival estimates was limited.

## 5. Conclusion

This is the first study of its kind in Central Asia to examine the burden of PD using a large-scale outpatient registry. This resulted in a significantly higher proportion of females in the cohort, highlighting the significant burden of PD in males and those aged 60 years and above. Additionally, further research is needed to explore the inverse association between hypertension and survival in the PD cohort. Despite limitations, this study is expected to contribute valuable knowledge to future research in developing countries.

## Figures and Tables

**Table 1:** Patient characteristics.

	Total (*n* = 10,125)	Female (6142; 60.66%)	Male (*n* = 3983; 39.34%)	*p* value
Sociodemographics				
Age category, *n* (%)				< 0.001
< 50 y.o.	625 (6.17%)	337 (5.49%)	288 (7.23%)	
50–59 y.o.	1807 (17.85%)	977 (15.91%)	830 (20.84%)	
60–69 y.o.	3591 (35.47%)	2143 (34.89%)	1448 (36.35%)	
70–79 y.o.	3141 (31.02%)	2041 (33.23%)	1100 (27.62%)	
≥ 80 y.o.	961 (9.49%)	644 (10.49%)	317 (7.96%)	
Ethnicity, *n* (%)				< 0.001
Kazakh	5157 (50.93%)	2958 (48.16%)	2199 (55.21%)	
Russian	3119 (30.80%)	2069 (33.69%)	1050 (26.36%)	
Other	1849 (18.26%)	1115 (18.15%)	734 (18.43%)	
Outcome				< 0.001
Living	8384 (82.80%)	5260 (85.64%)	3124 (78.43%)	
Died	1741 (12.20%)	882 (14.36%)	859 (21.57%)	
Charlson Comorbidity Index				< 0.001
0	420 (4.15%)	212 (3.45%)	208 (5.22)	
1-2	3810 (37.6%)	2205 (35.90%)	1605 (40.30%)	
3-4	4515 (44.6%)	2846 (46.34%)	1669 (41.90%)	
≥ 5	1380 (3.6%)	879 (14.31%)	501 (12.58%)	
Comorbidity				
Hypertension, *n* (%)				< 0.001
Yes	5862 (57.90%)	3824 (65.23%)	2038 (34.77%)	

**Table 2:** Association of covariates with all-cause mortality in PD patients.

Variables	Model 1. Unadjusted HR and 95% CI	*p* value	Model 2. Adjusted HR and 95% CI	*p* value
Age	1.04 (1.03–1.05)	< 0.001	1.05 (1.04–1.06)	< 0.001
Sex				
Female	1.0		1.0	
Male	1.52 (1.38–1.67)	< 0.001	1.62 (1.48–1.79)	< 0.001
Ethnicity				
Kazakh	1.0		1.0	
Russian	1.07 (0.96–1.19)	0.20	0.84 (0.75–0.94)	0.01
Other	1.16 (1.02–1.31)	0.02	0.97 (0.86–1.11)	0.72
CCI				
0	1.0		1.0	
1-2	1.13 (1.03–1.25)	0.01	1.26 (1.14–1.40)	< 0.001
3-4	1.24 (1.03–1.50)	0.03	1.30 (1.06–1.56)	0.01
≥ 5	1.85 (1.40–2.48)	< 0.001	2.10 (1.56–2.80)	< 0.001
Hypertension				
No	1.0		1.0	
Yes	0.79 (0.72–0.87)	< 0.001	0.66 (0.60–0.73)	< 0.001

*Note:* Model 1 unadjusted; model 2 adjusted for sex, ethnicity, CCI, and hypertension; variance inflation factor (VIF) = 2.01; Schoenfeld test = 0.79.

Abbreviation: HR = hazard ratio.

## Data Availability

Data are available on request from the authors.

## Supporting Information

Figure S1: flowchart of the cohort selection process from the Unified National Electronic Health System (UNEHS). Figure S2: crude and age-specific incidence and prevalence of PD (per 100,000). Figure S3: sensitivity analysis—exclusion of age-adjustment in CCI, outliers (A) and mortality rate across the study period (B). Figure S4: survival probability by sex, ethnicity, CCI, and hypertension (following propensity score matching (3690 hypertensive vs. 3690 nonhypertensive)). Figure S5: region-adjusted prevalence of PD in 2021. Table S1: age and sex-adjusted years of life lost due to premature death (YLL), years lived with disability (YLD), and disability-adjusted life years (DALY) for PD.
