# Supplementary material for: The Regional Burden of Parkinson's Disease in Kazakhstan 2014–2021: Insights From National Health Data
Source: Parkinsons Dis. 2025 May 1;2025:4317554. doi: 10.1155/padi/4317554 (PMC12061520; doi:10.1155/padi/4317554)
Supplement: Supporting Information — Additional supporting information can be found online in the Supporting Information section. [file 4317554.f1.docx]

Supporting material,

**The Regional Burden of Parkinson ’s Disease in Kazakhstan 2014–2021: Insights From National Health Data**

Authors: Ruslan Akhmedullin^1^, Arnur Gusmanov^1^, Gulnur Zhakhina^1^, Byron Crape^1^, Temirgali Aimyshev^1^, Yuliya Semenova^1^, Gaziz Kyrgyzbay^2^ and Abduzhappar Gaipov^1^.

Affiliation:

1. Department of Medicine, Nazarbayev University School of Medicine, Astana, Kazakhstan;
2. Department of Functional Diagnostics, RSE Medical Centre Hospital of the President's Affairs Administration of the Republic of Kazakhstan, Astana, Kazakhstan;

Corresponding author: Abduzhappar Gaipov, MD, Ph.D., Associate professor, Department of Medicine, Nazarbayev University School of Medicine. Address: Kerey and Zhanibek street 5/1, Postal code 010000, Astana city, Republic of Kazakhstan. Phone: +77172706297. Email: [abduzhappar.gaipov@nu.edu.kz](mailto:abduzhappar.gaipov@nu.edu.kz)

**This document contains**:

Figure S1. Flow chart of the cohort selection process from the Unified National Electronic Health System (UNEHS);

Figure S2. Crude and age-specific incidence and prevalence of PD (per 100,000);

Figure S3. Sensitivity analysis: Exclusion of age-adjustment in CCI, outliers (A) and mortality rate across the study period (B);

Figure S4. Survival probability by sex, ethnicity, CCI and hypertension (following propensity score matching (3,690 hypertensive vs 3,690 non-hypertensive));

Figure S5. Region-adjusted prevalence of PD in 2021.

Table S1. Age and sex-adjusted years of life lost due to premature death (YLL) and years lived with disability (YLD), and disability-adjusted life years (DALY) for PD;

**SUPPLEMENTARY MATERIALS.**

Figure S1. Flow chart of the cohort selection process from the Unified National Electronic Health System (UNEHS).


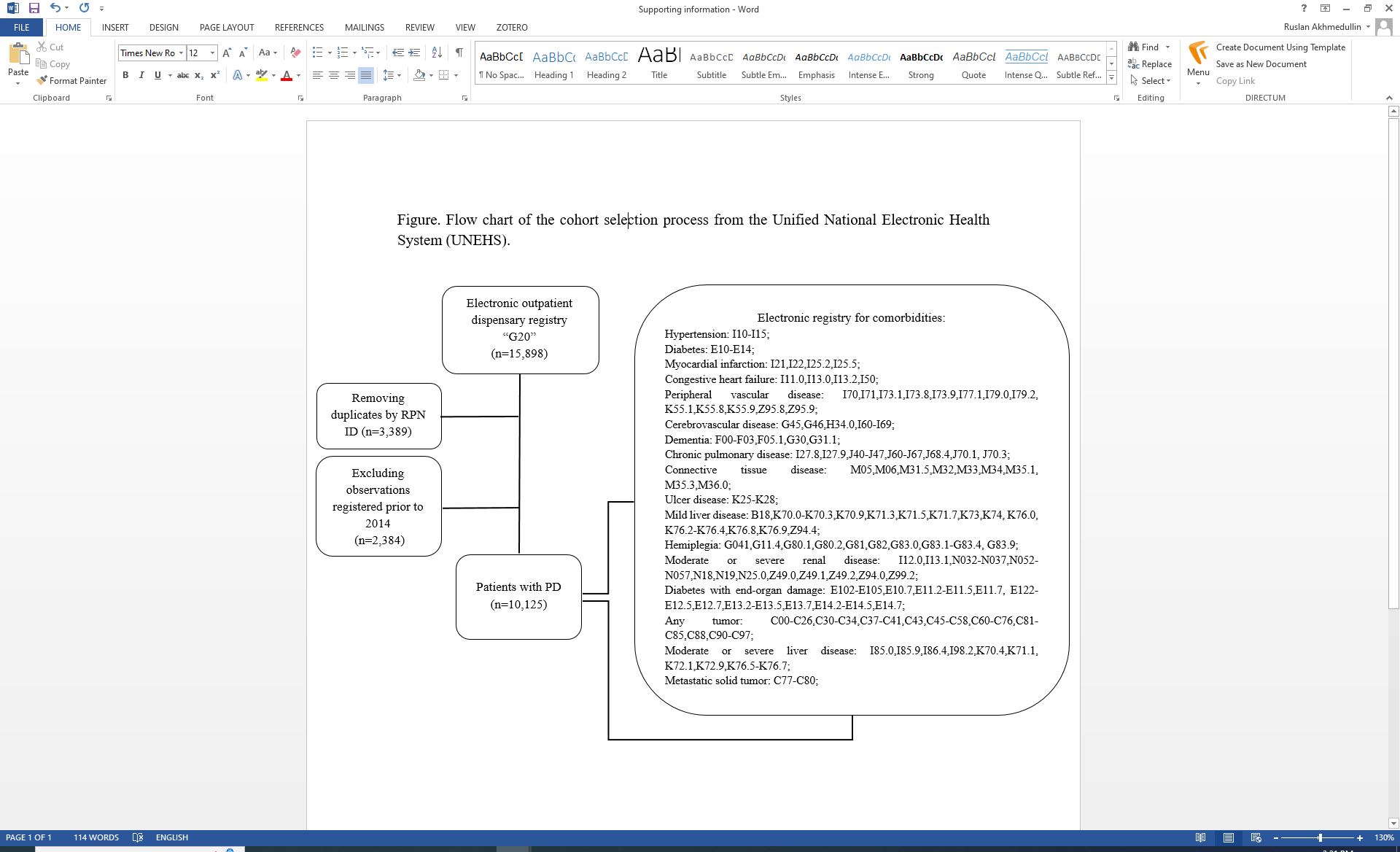


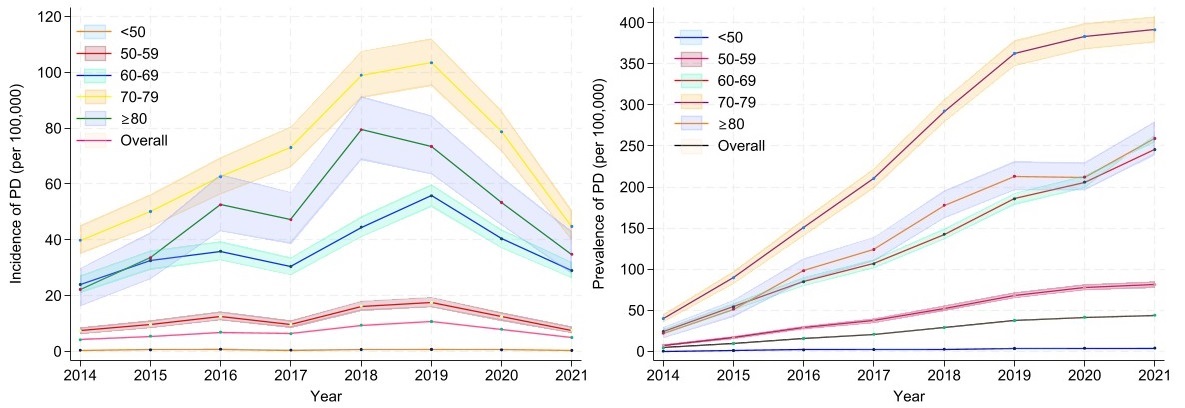
Figure S2. Crude and age-specific incidence and prevalence of PD (per 100,000).

Figure S3. Sensitivity analysis: Exclusion of age-adjustment in CCI, outliers (A) and mortality rate across the study period (B).


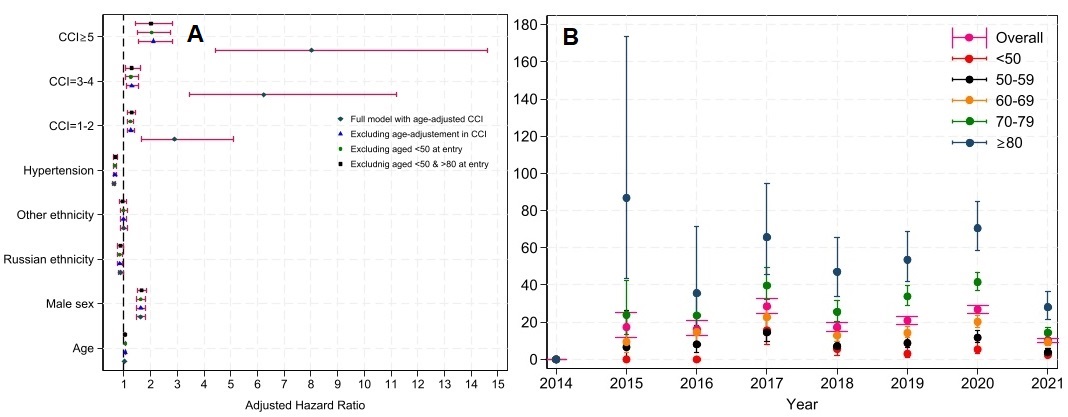


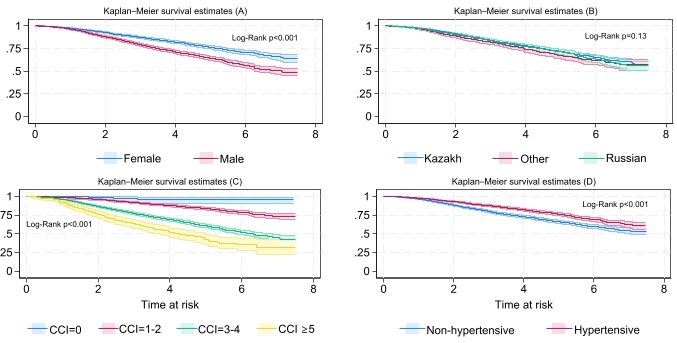
Figure S4. Survival probability by sex, ethnicity, CCI and hypertension (following propensity score matching (3,690 hypertensive vs 3,690 non-hypertensive)).

Figure S5. Region-adjusted prevalence of PD in 2021.


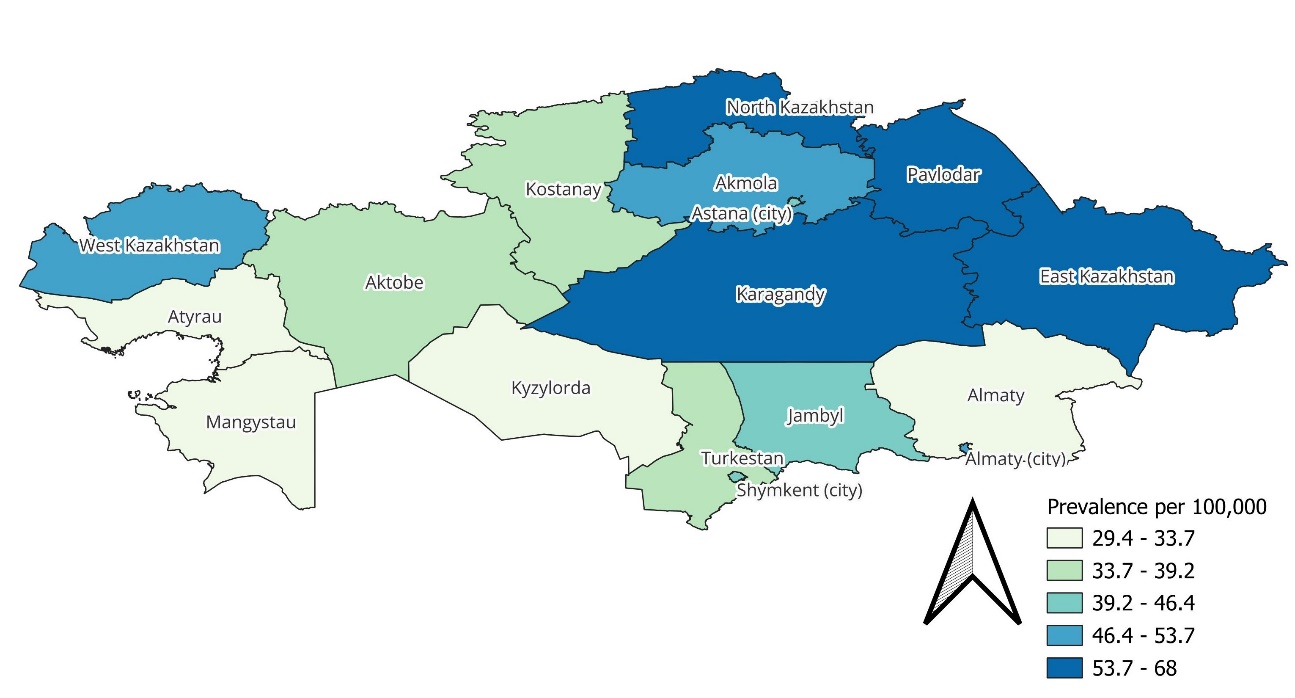


Table S1. Age and sex-adjusted years of life lost due to premature death (YLL) and years lived with disability (YLD), and disability-adjusted life years (DALY) for Parkinson’s disease.

| **Age-group** | **Life**  **expectancy** | **Female** | | | **Male** | | | **Total** | | |
| --- | --- | --- | --- | --- | --- | --- | --- | --- | --- | --- |
|  |  | **YLL** | **YLD** | **DALY** | **YLL** | **YLD** | **DALY** | **YLL** | **YLD** | **DALY** |
| ≤5 | 84.10 | 0 | 1.86 | 1.86 | 0 | 0 | 0 | 0 | 1.86 | 1.86 |
| 5 – 9 | 79.60 | 0 | 3.09 | 3.09 | 0 | 0 | 0 | 0 | 3.09 | 3.09 |
| 10 - 14 | 74.90 | 0 | 3.40 | 3.40 | 0 | 6.41 | 6.41 | 0 | 9.82 | 9.82 |
| 15 - 19 | 69.60 | 0 | 2.33 | 2.33 | 0 | 11.70 | 11.70 | 0 | 14.03 | 14.03 |
| 20 - 24 | 64.60 | 0 | 10.32 | 10.32 | 0 | 5.52 | 5.52 | 0 | 15.84 | 15.84 |
| 25 - 29 | 59.60 | 54.67 | 8.28 | 62.95 | 0 | 11.30 | 11.30 | 0 | 19.58 | 19.58 |
| 30 - 34 | 54.60 | 0 | 16.07 | 16.07 | 48.71 | 18.43 | 67.15 | 56.67 | 34.51 | 89.18 |
| 35 - 39 | 49.60 | 45.55 | 25.70 | 71.22 | 141.70 | 31.31 | 172.99 | 145.52 | 56.98 | 202.50 |
| 40 - 44 | 44.60 | 116.92 | 91.51  .541 | 208.43 | 121.56 | 63.15 | 184.71 | 175.78 | 154.66 | 330.44 |
| 45 - 49 | 39.60 | 390.03 | 162.46 | 552.50 | 390.11 | 140.61 | 530.73 | 415.46 | 303.10 | 718.56 |
| 50 - 54 | 34.60 | 641.38 | 344.27 | 985.65 | 651.14 | 299.87 | 951.01 | 1075.15 | 644.15 | 1719.30 |
| 55 – 59 | 29.60 | 1,193.0 | 523.05 | 1,716.05 | 1703.93 | 460.32 | 2164.25 | 1936.98 | 983.37 | 2920.35 |
| 60 - 64 | 24.60 | 1,637.89 | 788.24 | 2,426.14 | 2264.83 | 545.56 | 2810.40 | 3317.62 | 1,333.80 | 4651.42 |
| 65 - 69 | 19.60 | 2,214.79 | 976.50 | 3,191.29 | 2479.26 | 586.72 | 3065.98 | 4459.86 | 1,563.22 | 6023.08 |
| 70 - 74 | 14.60 | 1,868.90 | 787.97 | 2,656.86 | 2005.65 | 433.10 | 2438.71 | 4644.39 | 1,221.04 | 5865.43 |
| 75 - 79 | 9.60 | 1,530.41 | 822.0 | 2,352.36 | 1251.38 | 387.53 | 1638.92 | 3372.40 | 1,209.48 | 4581.88 |
| 80 - 85 | 4.60 | 299.60 | 316.96 | 616.56 | 233.57 | 151.10 | 384.65 | 1644.35 | 468.04 | 2112.39 |
| ≥85 | 1.60 | 0.77 | 104.51 | 105.29 | 1.29 | 46.09 | 47.38 | 44.89 | 150.61 | 195.5 |
| Total |  | 9,993.91 | 4,988.52 | 14,982.38 | 11,293.13 | 3,198.68 | 14,491.815 | 21,287.04 | 8,187.20 | 29,474.24 |
